# Gap-free genome assembly and comparative analysis reveal the evolution and anthocyanin accumulation mechanism of *Rhodomyrtus tomentosa*

**DOI:** 10.1093/hr/uhad005

**Published:** 2023-01-19

**Authors:** Fangping Li, Shiqiang Xu, Zitong Xiao, Jingming Wang, Yu Mei, Haifei Hu, Jingyu Li, Jieying Liu, Zhuangwei Hou, Junliang Zhao, Shaohai Yang, Jihua Wang

**Affiliations:** Guangdong Provincial Key Laboratory of Crops Genetics and Improvement, Crop Research Institute, Guangdong Academy of Agriculture Sciences, Guangzhou 510640, China; Guangdong Provincial Key Laboratory of Plant Molecular Breeding, State Key Laboratory for Conservation and Utilization of Subtropical Agro-Bioresources, South China Agricultural University, Guangzhou 510642, China; Guangdong Provincial Key Laboratory of Crops Genetics and Improvement, Crop Research Institute, Guangdong Academy of Agriculture Sciences, Guangzhou 510640, China; Guangdong Provincial Key Laboratory of Crops Genetics and Improvement, Crop Research Institute, Guangdong Academy of Agriculture Sciences, Guangzhou 510640, China; Guangdong Provincial Key Laboratory of Plant Molecular Breeding, State Key Laboratory for Conservation and Utilization of Subtropical Agro-Bioresources, South China Agricultural University, Guangzhou 510642, China; Guangdong Provincial Key Laboratory of Crops Genetics and Improvement, Crop Research Institute, Guangdong Academy of Agriculture Sciences, Guangzhou 510640, China; Guangdong Provincial Key Laboratory of Plant Molecular Breeding, State Key Laboratory for Conservation and Utilization of Subtropical Agro-Bioresources, South China Agricultural University, Guangzhou 510642, China; Guangdong Provincial Key Laboratory of Crops Genetics and Improvement, Crop Research Institute, Guangdong Academy of Agriculture Sciences, Guangzhou 510640, China; Rice Research Institute & Guangdong Key Laboratory of New Technology in Rice Breeding & Guangdong Rice Engineering Laboratory, Guangdong Academy of Agricultural Sciences, Guangzhou 510640, China; Guangdong Provincial Key Laboratory of Crops Genetics and Improvement, Crop Research Institute, Guangdong Academy of Agriculture Sciences, Guangzhou 510640, China; Guangdong Provincial Key Laboratory of Plant Molecular Breeding, State Key Laboratory for Conservation and Utilization of Subtropical Agro-Bioresources, South China Agricultural University, Guangzhou 510642, China; Guangdong Provincial Key Laboratory of Plant Molecular Breeding, State Key Laboratory for Conservation and Utilization of Subtropical Agro-Bioresources, South China Agricultural University, Guangzhou 510642, China; Rice Research Institute & Guangdong Key Laboratory of New Technology in Rice Breeding & Guangdong Rice Engineering Laboratory, Guangdong Academy of Agricultural Sciences, Guangzhou 510640, China; Guangdong Provincial Key Laboratory of Crops Genetics and Improvement, Crop Research Institute, Guangdong Academy of Agriculture Sciences, Guangzhou 510640, China; Guangdong Provincial Key Laboratory of Crops Genetics and Improvement, Crop Research Institute, Guangdong Academy of Agriculture Sciences, Guangzhou 510640, China

## Abstract

*Rhodomyrtus tomentosa* is an important fleshy-fruited tree and a well-known medicinal plant of the Myrtaceae family that is widely cultivated in tropical and subtropical areas of the world. However, studies on the evolution and genomic breeding of *R. tomentosa* were hindered by the lack of a reference genome. Here, we presented a chromosome-level gap-free T2T genome assembly of *R. tomentosa* using PacBio and ONT long read sequencing. We assembled the genome with size of 470.35 Mb and contig N50 of ~43.80 Mb with 11 pseudochromosomes. A total of 33 382 genes and 239.31 Mb of repetitive sequences were annotated in this genome. Phylogenetic analysis elucidated the independent evolution of *R. tomentosa* starting from 14.37MYA and shared a recent WGD event with other Myrtaceae species. We identified four major compounds of anthocyanins and their synthetic pathways in *R. tomentosa*. Comparative genomic and gene expression analysis suggested the coloring and high anthocyanin accumulation in *R. tomentosa* tends to be determined by the activation of anthocyanin synthesis pathway. The positive selection and up-regulation of MYB transcription factors were the implicit factors in this process. The copy number increase of downstream anthocyanin transport-related OMT and GST gene were also detected in *R. tomentosa.* Expression analysis and pathway identification enriched the importance of starch degradation, response to stimuli, effect of hormones, and cell wall metabolism during the fleshy fruit development in Myrtaceae. Our genome assembly provided a foundation for investigating the origins and differentiation of Myrtaceae species and accelerated the genetic improvement of *R. tomentosa.*

## Introduction

*Rhodomyrtus tomentosa* (Ait.) Hassk, called‘Taojinniang’ in Chinese, also known as rose myrtle, is a flowering plant in the family Myrtaceae, native to southern and southeastern Asia, including China, India, Philippines, Malaysia, and Sulawesi [1]. It is regarded as a medicinal herbal medicine effective in nourishing the blood system, resisting rheumatism, treating hematemesis, diarrhea and uterine bleeding in traditional Chinese medicine (TCM) [2]. Modern pharmacological investigations have identified a variety of pharmacological effects such as antibacterial, anti-tumor, anti-inflammatory and anti-oxidation in the metabolite extracted from *R. tomentosa* [3].

Fruit coloring and anthocyanins accumulation were a prominent features of *R. tomentosa* in myrtaceae. Based on the unique structural characteristics, anthocyanins possess various biological activities, including antioxidant, anticancer, anti-inflammatory, anti-artery atherosclerosis, anti-hypertensive, and antibacterial activities [2]. In recent decades, the research on the biological mechanism of anthocyanins has attracted global academic interest. The synthetic types and pharmacological effects of anthocyanins in myrtle were also preliminarily identified in previous studies [3]. However, few studies were published on the genetic basis of anthocyanins and other bioactive substances in *R. tomentosa*.

Molecular mechanism analysis was essential to understand the formation of anthocyanins and other bioactive compounds. Numerous types of research have shown that anthocyanin synthesis is a branch of flavonoid synthesis [4]. This pathway is regulated by various transcription factors represented by myeloblastosis (MYB) transcription factors [5]. The glycosylation and transport of anthocyanins also play an important role in fruit coloring [6, 7]. Nevertheless, the variation between myrtaceae species contributing to the unique characteristics of purple fruit in *R. tomentosa* remained unknown.

With the traditional basis of fruit types, the researchers of taxonomy divided the species of myrtaceae into two sub-families: Myrtoideae (fleshy-fruited) and Leptospermoideae (capsular-fruited) [8]. *R. tomentosa* with fleshy fruits was classified in sub-family Myrtoideae. Previous research on *Psidium guajava* (guava), another Myrtoideae species, indicated its fruit softening was promoted by starch degradation and cell wall activity [9]. Whether this trend is a common phenomenon among species in Myrtoideae clearly needs to be solved by more research in this sub-family.

Additionally, in the general view, the edible and medicinal parts were mostly focused on the fruit in *R. tomentosa*. In recent years, the effective medicinal components in leaves and stems of rose myrtle, such as rhodomyrtosone B and tomentosone C, have gradually attracted attention [10, 11]. However, a comprehensive and systematic study of medicinal flavonoids in *R. tomentosa* was still lacking due to the lack of genomic information.

Gap-free genomes provide the opportunity to identify genomic information as completely as possible, which is regarded as the ultimate goal of genome assembly. Previous research had released Gap-free genomes in multiple species including rice [12, 13], arabidopsis [14], and watermelon [15], but for Myrtaceae species, no gap-free genome has been reported.

In this study, we reported a telomere-to-telomere (T2T) gap-free genome if the elite *R. tomentosa* inbred line ‘LFSTJN-1’ with the combation of PacBio HIFI sequencing, Oxford Nanopore Technology (ONT sequencing as well as Hi-C techniques. We determined the major compounds of anthocyanins and their synthetic pathways in *R. tomentosa*. Comparative genomes provided possible regulated mechanisms for unique anthocyanin accumulation. Expression trend analysis and pathway identification further enriched the importance of starch degradation, signal transduction, and cell wall activity in fleshy fruit formation of Myrtaceae. Our genome assembly provides a foundation for investigating the origins and differentiation of Myrtaceae and accelerates the genetic improvement of *R. tomentosa.*

## Results

### The T2T gap-free reference genome for *R. tomentosa*

**Figure 1:**
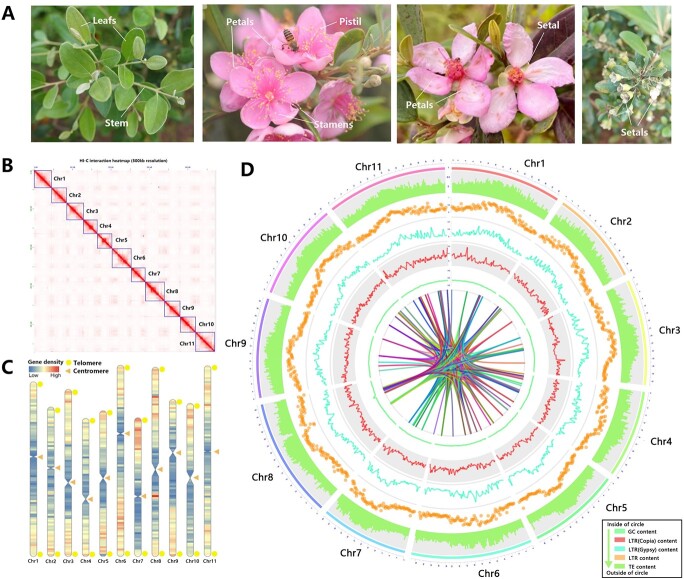
Display of images, genome assembly and genomic features of *R. tomentosa*. **A** The images of *R. tomentosa* including flower and leaves. **B** The HI-C interaction matrix based on the assembly. **C** Gene density and the distribution of telomeres and centromeres in *R. tomentosa* genome. **D** Genome characteristics of *R. tomentosa*, from inside to outside of the circle are distribution of genomic collinearity loci (homologous block of genome sequence), GC content, density of Long terminal repeats (LTR) Copia, density of LTR Gypsy, density of total LTR, density of total TE, respectively.

LFSTJN-1, an *R. tomentosa* cultivar widely planted in south China, was selected for T2T gap-free reference genome assembly (Fig. 1a). Genome survey indicated the genome of *R. tomentosa* was about 450.77 Mb with 0.29% heterozygosity (Fig. S1, see online supplementary material). Multiple sequencing platforms were used to develop a high-quality genome assembly for *R. tomentosa*. We generated ~33.40 Gb (∼74.08× coverage) HiFi reads using the PacBio sequel II platform and ~ 11.02 Gb (∼24.44× coverage) Oxford Nanopore Technology (ONT) ultra-long reads (Table S1, see online supplementary material). The N50 length of the HiFi reads and ONT reads were greater than 16 kb and 100 kb, respectively (Fig. S2, see online supplementary material). The preliminary assembly applied Hifiasm to HiFi reads and generated 482.47 Mb contigs with an N50 length of 29.87 Mb (Table 1). The assembly including 19 contigs with 471.22 Mb and N50 length of 39.17 Mb was generated by NextDenovo with ONT data and calibrated by NextPolish with HiFi reads as well as short reads from the illumina platform. The two assemblies were processed by 3D-DNA with HI-C data to correct assembly errors and remove redundant sequences. The HiFi-based assembly was implemented to fill in the gaps of the ONT assembly. After filling all remaining gaps, a 470.35 Mb gap-free genome of *R. tomentosa* was generated, containing 11 chromosomes with contig N50 length of 43.80 Mb. The number of chromosomes corresponded with the previous record (https://ccdb.tau.ac.il/). Finally, using seven base telomere repeats (‘CCCTAAA’) as a sequence query, we identified all 22 telomeres and constructed 11 T2T pseudomolecules of the *R. tomentosa* genome (Fig. 1C; Table S2, see online supplementary material).

**Table 1:** Statistics of genome assembly among Myrtaceae species

Statistics without reference	*R. tomentosa* (HIFI-contig)	*R. tomentosa* (ONT-contig)	*R. tomentosa* (Gap-free)	*P. guajava*	*E. grandis*
Number of contigs	269	19	11	44	4943
Number of contigs ($\ge$ 50 000 bp)	113	19	11	35	288
Largest contig	43 631 835	49 958 455	50 111 023	50 577 630	83 952 244
Total length	482 474 435	471 221 806	470 350 250	443 755 635	691 346 258
Total length ($\ge$ 50 000 bp)	477 256 938	471 221 806	470 350 250	443 448 379	651 047 186
N50	29 878 240	39 171 969	43 802 537	40 370 300	57 472 304
L50	7	6	5	5	5
GC (%)	40.63	40.56	40.56	39.48	39.29
# N’s (Mismatches)	0	0	0	2900	50 906 790
# N’s per 100 kbp (Mismatches)	0	0	0	0.65	7363.43

Further analysis with TRF identified two types of major candidate centromere tandem repeats with monomer length of 422 bp and 153 bp in *R. tomentosa* genome (Figure S3, see online supplementary material). One putative centromere was identified for each of the 11 pseudochromosomes, with the length ranging from 0.35 Mb to 3.49 Mb. The gene density in *R. tomentosa* increased from the centromeres toward the chromosome ends, which was corresponding with the distribution in multiple species (Fig. 1C; Table S3, see online supplementary material).

### Quality assessment of *R. tomentosa* assembly

To confirm the genetic background of *R. tomentosa* assembly, multiple data and methods were implemented. From a sequence point of view, an interaction matrix from generated HI-C short-reads library indicated 11 chromosomes were shown to be fully and reasonably assembled (Fig. 1B), whose numbers were consistent with previous records. In the meantime, the alignment of short reads and HIFI reads data utilized in the survey and assembled showed a mapping rate of ~99.96% and ~ 99.93%. From the genetic perspective, BUSCO evaluation base on eudicots_odb10 as well as embryophyta_odb10 indicated that 97.7% (2067 out of 2284) and 99.0% of the core conserved plant genes were completely found in the assembly (Table S4), respectively. Moreover, we successfully mapped the 92.4% to 96.5% of RNA-Seq datasets generated from different tissues and developmental stages to the assembly. The completeness test of long terminal repeat (LTR) showed a 16.16 LTR assembly index (LAI) values for the assembly, which was similar to the gap-free assembly such as *Actinidia chinensis* cv. ‘Hongyang’ and higher than that of *P. guajava* (~9.38) [9, 16]. These data suggested a high-quality *R. tomentosa* genome assembly.

### Genome annotation and repeat sequence recognition

The combination of* de novo*, homology-based evidence suggested the repeat sequences accounted for 50.92% (239.51 Mb) of the assembled genome ,in which 190.23 Mb (79.42%) of the sequences belonged to long-terminal repeat retrotransposons (Fig. 1d; Table S5, see online supplementary material). The repeated masked genome was utilized to predict gene structures by combining the results of *de novo*, homolog-based, and transcriptome-based predictions. Finally, 33 382 protein-coding genes were predicted in our assembled genome, with an average coding sequence size of 1483.1 bp and an average of 4.92 exons per gene. The completeness of annotation was assessed through BUSCO. Approximately 93.2% of complete gene elements were detected in the gene set of *R. tomentosa*, which elucidated that most conservative genes were predicted exactly and reflected the high reliability of prediction results (Table S6, see online supplementary material).

### Genome evolution

**Figure 2:**
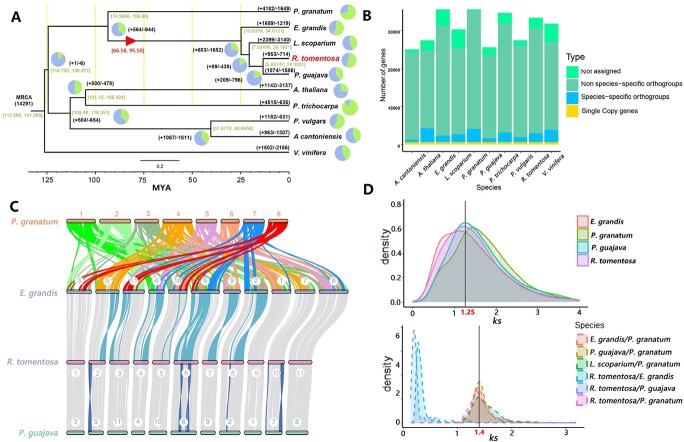
Phylogenetic and collinearity analysis among *R. tomentosa* and other species. **A** Phylogenomic analysis and gene family distribution of *R. tomentosa* and another nine plant species. The values on the nodes represent the divergence time from now (unit: million years ago, MYA); the red triangle shows the WGD events in Myrtaceae. **B** Gene distribution of all gene families in 10 species. **C** Gene-based genome colinear comparison among three species of the Myrtaceae and *P. granatum.***D** Synonymous substitutions per synonymous site (ks) plot of WGD events detection of species and *P. granatum*.

Phylogenetic and evolutionary event analysis were necessary to clarify the status of *Rhodomyrtus* in Myrtaceae. Time-scaling phylogenetic trees based on 617 single copy orthologues genes indicated that the divergence time between *Rhodomyrtus* and *Punica* was about 14.37 million years ago (MYA) while 953 and 714 gene families showed expansion and contraction in *R. tomentosa,* respectively (Fig. 2A and B). Collinearity analysis among three Myrtaceae species as well as * Punica granatum* (pomegranate) showed complete and continuous collinearity. Further identification of chromosomal variation revealed that the recombination of pomegranate chromosomes played an important role in the speciation of Myrtaceae. Meanwhile, more chromosomal inversions were detected in *Eucalyptus grandis* than *P. guajava* in the separate comparison with *R. tomentosa*, which may drive the differentiation within Myrtaceae (Fig. 2C).

The distribution of synonymous substitution sites (*Ks*) for paralogous genes in Myrtaceae species including *R. tomentosa* showed a peak at *Ks* ≈ 1.25, which revealed that *R. tomentosa* shared acommon recent whole-genome duplicated event (WGD) with other myrtaceae species(Fig. 2d). Collinearity analysis also indicated that *R. tomentosa* retained the characteristic of ancestral polyploidy similar with other myrtaceae species including *P. guajava* (Fig. S4, see online supplementary material).

Based on the distribution of Ks, we further determined the age of common WGD events in Myrtales species. We further inferred an average *Ks*/year rate about 6.55 × 10^−9^ to 9.39 × 10^−9^ (confidence interval of 95%) in Myrtales based on the peaked at *Ks* ≈ 1.4 detected in orthologous between *P. granatum* and Myrtales species. This data suggested that the WGD events in Myrtaceae tended to occur 66.58–95.50 MYA (Fig. 2a).

### Gene expression patterns among organs and fruit softening related metabolism in *R. tomentosa*

The growth and activity of life cannot be separated from the gene expression in plants. To explore the gene expression patterns in the organs of *R. tomentosa,* a weighted gene correlation network analysis (WGCNA) was implemented using 25 038 expressed genes in ten types of samples from various organs and developmental stages. A thresholding power of eight was selected, which was the lowest power that properly fit the scale-free topological index (Fig. S5B, see online supplementary material). A total of 25 co-expression modules including 12 042 genes were revealed after the merged dynamic analysis while the rest of the genes were divided into Module Other (with grey color) (Table S7, Fig. S5c, see online supplementary material).

**Figure 3:**
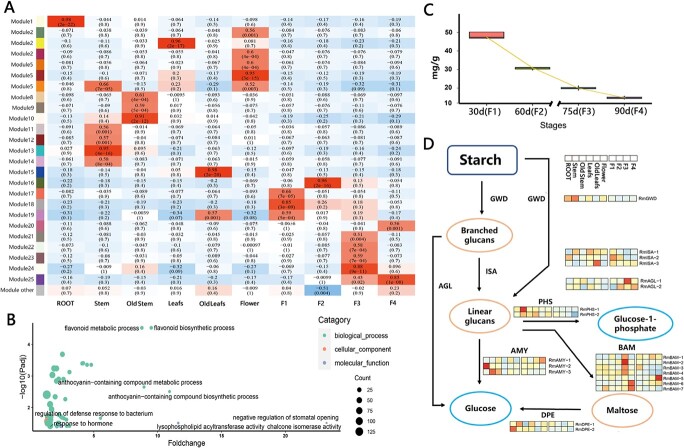
Gene expression patterns in different tissue as well as starch degradation pathway in *R. tomentosa.***A** Heatmap showing the correlation between modules and various tissues and developmental stages of *R. tomentosa*. The correlation coefficient between a given module and samples was indicated by the color of the cell and the text inside the cell (upper number is the value of Pearson correlation coefficient (*r*), and lower number is the *P*-value). Red and blue indicate positive and negative correlations, respectively. **B** The GO enrichment of the genes in module24; the ontologies with adjusted *P* < 0.05 was displayed while the ontologies mentioned in this research were texted: the detail was given in Table S12 (see online supplementary material). **C** The variation of starch content during fruit development of *R. tomentosa,* the x-axis represents the time after flowering and the corresponding period of fruit development (F1–F4). **D** Starch degradation pathway and the corresponding gene expression in *R. tomentosa*.

Previous research on *P. guajava* indicated the process of fruit softening and ripening was associated with cell wall activity and starch degradation. *R. tomentosa* was a good material to further explore the mechanism of fleshy fruit development in Myrtaceae. Correlation analysis detected the highly closely related co-expression modules (*r* > 0.8) of organs and developmental stages of samples (Fig. 3a). Further gene enrichment indicated the pathway related to cell wall activity was enriched in module16, which was closely related to F2 (*r* = 0.96). The Gene Ontology (GO) term of hormone response was enriched in the F3 related module (module24; *r* = 0.88; Fig. 3B) (Fig. S6, see online supplementary material). Gene enrichment for external response and stress was detected at all stages of fruit development. The regulation of cell wall degradation by hormones has been reported in a variety of fruit ripening studies including *P. guajava*, the species within the same subfamily as the published genome [9]. At the same time, the great influence of external response external response on fleshy fruit development was widely confirmed [17].

Besides, similar to *P. guajava*, the starch content of *R. tomentosa* decreased as the fruit develops (Fig. 3c). Based on Kyoto Encyclopedia of Genes and Genomes (KEGG) annotation, we identified 20 starch degradation related genes belonged to 7 families in *R. tomentosa*. The expression analysis showed that the gene related to starch metabolism process (GWD/ISA) was mainly expressed in the F1 stage (Fig. 3d; Fig. S7, see online supplementary material). Multiple highly specific expressed gene copies involved in metabolic of monosaccharides or polysaccharides were identified in F3 and F4 including *RmAGL-1*, *RmAGL-2*, *RmAMY-1,* and *RmBAM-5*. Correspondingly, the genes related to glycan metabolism were enriched in module25 which was closed related to F4 (*r* = 0.83; Fig. 3A)(Fig. S6, see online supplementary material).

In addition, those genes involved in starch degradation were also highly expressed during leaf or Astem. senescence. This is consistent with the process of organ aging in multiple species.

### Metabolites and gene expression patterns related to coloring and anthocyanin synthesis in *R. tomentosa* fruit development

**Figure 4:**
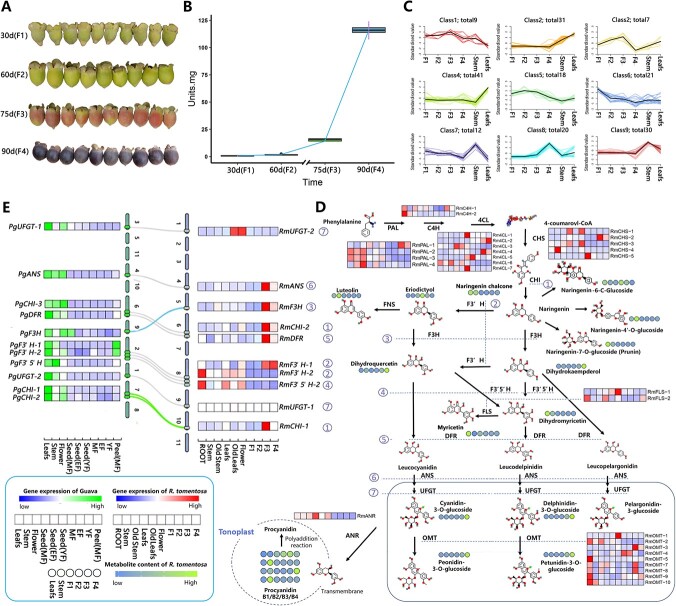
Metabolite abundance clustering and flavonoid (mainly for anthocyanin branch) compound synthesis pathway. **A** Images for *R. tomentosa* fruit at various stages of fruit development. **B** Trend of total anthocyanin content during fruit development of *R. tomentosa*. **C** Clustering of metabolites abundance based on K-means. **D** Trends of anthocyanin content during fruit development of *R. tomentosa*. **E** Flavonoid synthesis pathway, distribution of metabolite content corresponding gene expression, locus and collinearity of the orthologous genes between *R. tomentosa* and *Psidium guajava*.

The coloring during fruit ripening is an indicative feature of *R. tomentosa* (Fig. 4a). Morphological observation and determination of total anthocyanin content showed that the anthocyanin content in fruits of *R. tomentosa* increased sharply during F3 to F4, and the fruit color also changed to purple (Fig. 4a and b). Accordingly, the genes in module24 were highly enriched in the pathways related to flavonoid synthesis and metabolism, especially anthocyanin synthesis and metabolism (Fig. 3b).

In order to investigate the mechanism between fruit coloring and flavonoid accumulation in *R. tomentosa,* metabolomic research for flavonoids was implemented. Metabolite assays with UP-MS detected 189 flavonoids in six types of samples from three organs of *R. tomentosa.* These metabolites were divided into nine abundance clusters (Fig. 4c; Table S8, Fig. S8, see online supplementary material). We spotlighted the compounds that accumulated in mature fruit (cluster8), which consist of 20 compounds, including anthocyanin glycosides, epicatechin, and quercetin. Anthocyanins in ripe fruit of *R. tomentosa* (cluster8) mainly existed in the form of four glycosides, including anthocyanin-3-O-glucoside, fetosidin-3-O-glucoside, paeoniflorin 3-O-glucoside, and paeoniflorin 3-O-glucoside. Anthocyanins from geraniums was too low to be detected in fruit.

To investigate the relationship between co-expression modules and metabolomic data, we correlated the content and accumulation rate of the metabolites in fruit with the gene expression in various modules, respectively. The highest correlation was detected between module25 and metabolite content in cluster8 (Fig. S9, see online supplementary material). Functional analysis elucidated these genes mainly enriched in the 19 Go terms, including three terms related to vacuole (Fig. S6b, see online supplementary material). Previous studies have shown that fruit color changes were closely related to the transport of metabolites to vacuoles [18]. Whether the accumulation of anthocyanin in F4 was related to the genes in this module was worth further exploration. More importantly, the expression of genes in module24 was closely related to accumulation rate metabolite in cluster8 (average *R* = 0.873). This was corresponding to the GO enrichment results of F3-related modules (module24), which suggested the genes in module24 tended to play an important role in anthocyanin accumulation and coloring in fruit of *R. tomentosa*.

We further investigated the expression patterns and metabolite fluctuation based on the anthocyanin biosynthetic pathway documented in KEGG. Based on the records, a total of eight types of gene were regarded as encoding the core enzymes in anthocyanin biosynthesis. During fruit coloring, although the gene expression trend in module25 was closely related to the differences in the content trends of 20 metabolites and total anthocyanins, only one gene, *Rm4CL-2,* was detected in the anthocyanin synthesis pathway. In contrast, at least one copy in six types of enzymes (4CL, CHS, CHI, F3H, ANS, DFR) in the anthocyanin synthesis pathway were clustered in module24 which was upregulated in F3. The gene copies (*RmPAL-1*, *RmC4H-1*) showing as upregulated in F3 during fruit development were also detected in the two enzymes (PAL, C4H). In the core process of anthocyanin synthesis (from Naringenin chalcone to anthocyanins), all recognized core enzymes including *RmCHI-1, RmCHI-2, RmF3H, RmDFR*, and *RmANS* were highly expressed in the F3 stage. This trend corresponded to the sharp change and increase of fruit color and anthocyanin content in *R. tomentosa* from F3 to F4 (75 to 90 days after flowering; Fig. 4c and d).

### Fruit coloring and anthocyanin accumulation in the Myrtaceae

The evolutionary footprint of natural selection behind the unique peel color of *R. tomentosa* in myrtaceae remained elusive. *P. guajava*, another fleshy-fruited myrtaceae species with green peel and the pulp of fruit [9], was the most suitable specie for comparative analysis. Unlike *R. tomentose,* transcriptome analysis detected the genes involved in anthocyanin core synthesis were not elevated but downregulated in *P. guajava* fruit (with negative correlation of the orthologous genes in *R. tomentose*; Table S9, see online supplementary material). This difference of expression fluctuation suggested that anthocyanin synthesis activity was not enhanced in *P. guajava* fruit, which was consistent with the undertint color of the *P. guajava* fruit (Fig. 4D and E) [9].

**Figure 5:**
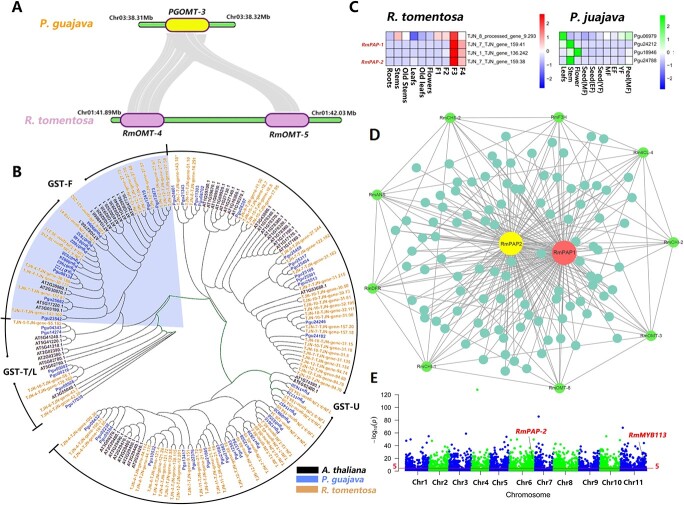
Analysis of gene copy numbers, MYB and GST gene family phylogenetic, MYB differentially expressed gene as well as positive selection gene. **A** An extra copy of the OMT gene in Chr1 in *R. tomentosa* compared to the orthologous gene in *P. guajava*. **B** Gene family analysis showed that compared with *P. guajava*, the GST family genes in *R. tomentosa* showed an increased copies number (76 > 51). **C** The expression of MYB gene in module24 in *R. tomentosa* and its orthologous gene in *P. guajava*. **D** The correlation network in module24 highly related to *RmPAP-1* and *RmPAP-2*. The nodes of gene in anthocyanin biosynthesis pathway were colored with green, the transparency of the edges indicates the weight value between two genes. The size of the node represents the number of connected genes. **E** Positive selection genes and distribution of *R. tomentosa.*

Besides, comparative research of downstream pathway of anthocyanin synthesis between these two species reported a copy number variation (CNV) of the OMT genes required for glycosylation of anthocyanin. Two gene copies (*RmOMT-4*/*RmOMT-5*) resulting from tandem repeated expansion were located at the end of chromosome 1 (Fig. 5A). These gene copies have high sequence homology and structural similarity with the orthologous gene (Pgu20700) in *P. guajava*. Expression analysis showed that the expression of *RmOMT-4* was specifically increased in fruit while *RmOMT-5* was also expressed at all stages of fruit ripening, but the highest expression was found in the root (Fig. 4d). In the meantime, the transportation and accumulation of anthocyanin glycosides greatly affect the color of plants. GST super gene family was previously reported to participate in the transport process of anthocyanin [18]. Based on KEGG annotation, we detected more GST genes in *R. tomentosa* than in *P. guajava *(76 > 51; Fig. 5B). Subfamily analysis based on phylogenetic relationships illustrated that there were more GSTU subfamily genes in *R. tomentosa* than *P. guajava* (56 > 32; Fig. 5B). This subfamily was proven to participate in anthocyanin transport [19]. The relationship between the CNVs and potential difference in anthocyanin glycosylation as well as the transportation, which further affects the fruit coloring in *R. tomentosa* and *P. guajava* is worthy of further investigation.

### Potential effects of positive selected MYB genes on anthocyanin synthesis in *R. tomentosa*

Further analysis of the MYB gene family in this research detected a similar number of MYB genes in *R. tomentosa* and *P. guajava* (251/249) (Fig. S10, see online supplementary material). Among them, the expression of 4 MYB genes was clustered in module24 in WGCNA. In contrast, a similar specific period of high expression in fruit was not detected in the homologous gene of these four genes in *P. guajava* (Fig. 5C). Phylogenetic analysis indicated two of the four genes (*RmPAP1* and *RmPAP2*) were clustered with *PAP1(MYB75), PAP2(MYB90)* as well as *AtMYB113* in *A. thaliana* (Fig. S10, see online supplementary material)*,* whose high expression had been proven to promote anthocyanin synthesis. The co-expression networks of these two MYB transcription factors contained nine genes in anthocyanin synthesis pathways (Fig. 5D).

In the meantime, the detection of positive selected genes among *R. tomentosa* and other Myrtaceae species elucidated 2432 genes were positively selected in *R. tomentosa.* These genes covered 32 MYB transcription factors including *RmPAP-2 and RmMYB113.* This difference in the interspecies expression trend and positive selection in evolution of the positive MYB regulator of anthocyanins tended to result in fruit coloring of *R. tomentosa* (Fig 5E; Table S9, see online supplementary material). The remaining positively selected MYB transcriptionfactors tended to participate in multiple biological process of plant including pollen development (MYB80), seed germination (MYBC1), and epidermal development (ETC2), respectively (Table S10, see online supplementary material).

### The medicinal activity of flavonoids in leaves and stems of *R. tomentosa*

Previous studies have shown that a variety of flavonoid metabolites with medicinal value were extracted from the leaves or stems of corresponding medicinal plants. In this research, the content of dihydromyricetin, an anthocyanin precursor, was significantly elevated in plant leaves while its fluctuation was different from that of anthocyanin in the downstream. Dihydromyricetin was also reported to be a direct precursor of another medicinal flavonoid, myricetin, whose content was also elevated in leaves. Correlation analysis showed that the expression of **RmF3' 5' H*, RmFLS* required for dihydromyricetin formation and myricetin transformation and was closely related to the content fluctuation of its metabolic products, respectively (Table S11, see online supplementary material; Fig. 4D). This suggested that more dihydromyricetin in leaves tended to participate in the accumulation of myricetin. Further investigation detected the similar mechanism in multiple flavonoid metabolites with medicinal value, such as luteolin and quercetin, which have high content in stem and leaf, respectively. This similar distribution pattern of medicinal metabolites was also found in herbal tea with a great utilization potential [20]. More experimental evidence and research is necessary for the verification and utilization of the pattern in *R. tomentosa* (Fig. 4D).

In addition, proanthocyanidins, an anthocyanins-like antioxidant bioactive substance, was detected with four isomers of type B (B1–B4) in *R. tomentosa*. Interestingly, the content of two isomers (B2/B4) was elevated only in the fruit of F4, while the remaining two were only highly detected in the leaves. The hidden functional variation behind the content differences of these isomers in different organs deserves further investigation (Fig. 4D).

## Discussion

Based on the previous statistics, above 3000 species in the Myrtaceae family are distributed predominantly in tropical and subtropical regions of the world [21]. *R. tomentosa,* one of the most iconic species in this family*,* is an evergreen shrub of the genera *Rhodomyrtus*, commonly found in east and southeast Asia [3]. Genomic resources with high quality and representativeness can facilitate molecular breeding and evolutionary studies of *R. tomentosa* as well as Myrtaceae. Here, by combining PacBio HiFi and ONT data and Hi-C data, a Gap-free telomere-to-telomere *R. tomentosa* genome was released in this research. The completed genome assembly will undoubtedly serve as a benchmark for further research in this species and Myrtaceae. The information of genome, metabolite abundance and gene expression patterns released in this research will also be a valuable genetic resource for molecular breeding and improve post-harvest storage strategies.

Anthocyanins and other flavonoids in fruits and vegetables protect against a variety of diseases, especially cardiovascular disease, and some types of cancer. At the same time, Anthocyanins were proven to significantly improve vision when administered to humans and animals [22]. The previous research about vital medicinal and nutritional properties has elucidated that flavonoid compounds, especially anthocyanins, are important bioactive substances in the fruit of *R. tomentosa* [3]. This is consistent with the high expression of the corresponding pathway synthetic genes, which also promote the coloring of *R. tomentosa* fruit. Metabolic research revealed the process was attributed to the accumulation of four types of anthocyanins*.* The accumulation of anthocyanins from geraniums was not detected in fruit because of an extremely low level of content, which was in line with previous quantitative studies [2].

High anthocyanin synthesis and accumulation often result from the high expression of gene encoding the core enzymes in the anthocyanin synthesis pathway (CHI, F3H, F3' H, DFR, ANS) [23]. Analysis of gene expression and metabolite fluctuation showed that this pathway was activated in F3 of *R. tomentosa*. Previous research proved that this mechanism was widespread and regulated by various transcription factors represented by genes from the MYB family [5]. Comparison of pathways and expression trends between *R. tomentosa* and *P. guajava* indicated significant differences in the expression trend of core genes related to anthocyanin synthesis in these two species, which was consistent with the color difference of their fruits. Further analysis of gene family and gene expression suggested this divergence between the two myrtaceae species resulted from the positive selection as well as various expression of multiple MYB transcription factors including PAP1, PAP2, MYB113, and MYB114, which had been proven to promote anthocyanin synthesis in *A. thaliana* [24, 25]*.* Similarly, the regulated mechanisms have been detected across multiple species, such as *Daucus carota* (carrot) and *Raphanus sativus* [26, 27]. More comparative genomic and physiological experiments are needed to prove whether it is a universal and important pathway responsible for the differences in anthocyanin accumulation among species.

In addition, glycosylation and transportaion are necessary to accumulate anthocyanins and color changes in plant organs. Genes from the OMT and GST family were the key factors in this process [6, 7]. Previous research had reported that the loss of GST family genes can seriously affect the accumulation of anthocyanins in flowers and peel of peach, which resulted in the white flowers and fruit peel [28]. In this research, the expression increased during fruit ripening was detected in copies of *RmOMT* in *R. tomentosa.* Compared with *P. guajava*, the gene copy number of the OMT and GST family showed as increased in *R. tomentosa.* CNV-affected morphological characteristics, such as disease resistance in humans or grain size in rice [29, 30], were frequently reported. Whether the CNVs found in this study had influence on anthocyanin metabolism in *R. tomentosa* is worthy of further investigation.

Fleshy fruit ripening in myrtaceae was accompanied by starch degradation and cell wall lysis [9]. The optimizing of seed dispersal depends on fruit ripening, which is controlled by a complex network of transcription factors and genetic regulators. This regulatory process is closely related to the action of plant hormones. The RNA-Seq analysis suggested that this mechanism also contributes to fruit development in *R. tomentosa*. This corresponds to the fruit softening process of *P. guajava*, another myrtaceae species of flesh fruit. Similar mechanisms were detected in previous research of fruit development such as mango (Anacardiaceae), strawberry (Rosaceae), and pear (Rosaceae) [9].

## Conclusion

Here we presented the first gap-free T2T genome in Myrtaceae family, *R. tomentosa* genome, as well as detailed genomic information. We determined the major compounds of anthocyanins and their synthetic pathways in *R. tomentosa*. The pattern analysis of gene expression and pathway identification further provided the new insights into fleshy fruit development in Myrtaceae. Comparative genomic and gene expression analysis provided possible mechanisms for anthocyanin accumulation and coloring in fruit. Our genome assembly represents a foundation for investigating the origins of fleshy fruits in Myrtaceae and for accelerating genetic improvement of *R. tomentosa*.

## Materials and methods

### Plant sample collection and sequencing of *R. tomentosa*

Root, stem, leaf, flower, and fruit tissues of ‘LFSTJN-1’, a popular cultivar of *R. tomentosa*, were collected from the Hui Zhou City (Boluo District, 23.238691°N, 114.044166°E) in Guangdong Province, China (Fig. 1A). High-quality genomic DNA was extracted using a QIAGEN Genomic Kit. The integrity of the DNA was checked by 0.75% agarose gel electrophoresis. DNA purity and quantity was detected by Nanodrop (OD260/280 = 1.8–2.0, OD260/230 = 2.0–2.2) and Qubit, respectively.

The transcriptome and metabolome samples covered ten and six periods of five tissues, respectively (Table S1, see online supplementary material).

### Hi-C experiment

Hi-C libraries of fresh young leaves were constructed with NEBNext Ultra II DNA library preparation kit and DpnII enzyme (Ipswich, MA, USA). The Illumina NovaSeq 6000 platform was utilized in sequence of Hi-C libraries. A total of 59.45 GB of cleaning data was generated in this process (Table S1, see online supplementary material).

### Genome survey, chromosome assembly construction, and gap filling

The genome size and heterozygosis of *R. tomentosa* were assessed by the software jellyfish and Genomescope [31]. For the PacBio assemblies, The Pacbio reads generated form CCS mode (HiFi reads) were assembled using software Hifiasm with the default parameters [32]. NextDenovo (https://github.com/Nextomics/NextDenovo) was implemented to assemble the ONT reads with the parameters: genome_size = 460 M, read_cutoff = 50 000, seed_cutoff = 55 959, seed_depth = 45. Hi-C data was used to separate the chimeric, which were processed by 3D-DNA and visualized by juicerbox [33, 34]. The HiFi-based assembly was implemented to fill the gaps of the ONT-assembled reference.

### Repeat element identification, gene prediction, and function annotation

Pipeline EDTA was implemented and used for repeated elements identification and masking. The masked genomic sequences were utilized in further gene prediction [35].

Gene prediction of the genome was accomplished by integrating *de novo* prediction, homologous protein evidence and EST evidence using pipeline Maker [36]. Software Augustus was utilized in *de novo* gene prediction while hisat2, stringties and PASA were implemented to generate EST evidence with 186.71Gb RNA-seq data [37]. Homologous protein sequences from *E. grandis* and *P. guajava* were imported into the exonerate module to generate homologous evidence. PASA also was utilized in the spliced site adjustment and UTR prediction.

Functional annotation was achieved by using NCBI BLAST with cutoff e-values of 1e-5 to compare predicted proteins against public databases. The BLAST results of Best-hit were considered as the gene functions. The annotation of GO and KEGG were obtained using EggNOG as well as KAAS [38]. The Telomere detection and centromere localization were processed by the combination of TRF and CD-HIT based on previous research [16, 39, 40].

### Genomic assembly quality assessment

Multiple methods were implemented to assess the accuracy and completeness of the assembled genome. Firstly, we calculated the basic indicators of chromosome length and genome assembly integrity represented by N50 and LAI score [41]. Besides, the paired-end short reads as well as HIFI reads were mapped to the genome using BWA-MEM while the RNA-seq data was aligned with hisat2 to evaluate its completeness. From the genetic perspective, Benchmarking Universal Single-Copy Orthologous (BUSCO) was implemented to assess the integrity of genome with database eudicots_odb10 and embryophyta_odb10 [42].

### Phylogenetic analysis, estimation of divergence time and gene family analysis

The gene orthologues including the single-copy orthologue genes of 10 species were determined by OrthoFinder2 [43]. The alignment of coding DNA sequences (CDS) of single-copy orthologues was finished by MAFFT with option -maxiterate 1000 [44]. The phylogenetic tree among species was deduced by software RaxML in GTRGAMMA model and 100 bootstrap with Maximum likelihood (ML) method [57]. The MCMCtree module of software PAML was implemented to determine the divergence time of each tree node [45]. Phylogenetic processes were calibrated using various fossil records. Molecular divergence times were estimated by placing soft boundaries on the split nodes using records from the timeree database (http://timetree.org/). Expandation and contraction of gene families were identified for each divergence node and species using the program CAFE with default parameters [46].

### Collinearity, positive selected genes, and WGD detection

The python version of MCScanX (JCVI) was used to identify the collinearity block [47] between myrtaceae species and *P. granatum* [9, 48, 49]. We performed multiple-sequence alignment to extract the conserved paralogs of the protein sequences of this data by using Orthofinder2 (e-value ≤1 × 10–5) [43]. The 3372 single copy orthologous generated by this process were used to identify the phylogenetic relationships between species for the detection of positive selected genes in *R. tomentosa*. Codeml module of PAML was implemented to calculate the selection pressure of the branch of *R. tomentosa* with a branch-site model [45]. Subsequently, the significance of positive selection genes was verified by the Chi-square test (*P*-value <1e-5).

For the detection of WGD, WGDI was used to identify and extract collinear intervals and corresponding gene pairs. The calculation of synonymous substitutions per synonymous site (*Ks*) with the method algorithm NG86 was implemented to predict the WGD events [50]. The ages of WGDs were estimated based on the formula: Divergence time = *Ks*/ (2 × *r*) [58, 59], we inferred *r* (plant average *Ks*/year rate) in Myrtales via *Ks* distributions of paralogous genes.

### Transcriptome data processing

The alignment of RNA-seq data was processed by pipeline Hisat2, while the Samtools was used in data sorting and conversion of file formats. The gene expression statistics were generated by the software FeatureCounts [51]. The R module DESeq2 was implemented to data normalize and identify the differentially expressed genes (DEGs) [52]. The transcriptome data of *P. guajava* organs and developmental stages were obtained from NCBI with Bioproject PRJNA631442 [9].

### WGCNA and correlation analysis

WGCNA was implemented to identify gene modules related to organs in *R. tomentosa* [53]. First, we clustered the samples based on the gene expression levels. Then the TOMsimilarity module and PickSoftThreshold functions of the software package were utilized to generate the coexpression similarity coefficient between genes and process of weight. This correlation was further translated into the association between genes. We used the absolute value >0.8 and *P*-value <0.001 to identify gene modules that were significantly correlated with organs. The remaining details were based on the protocol in previous research [54].

The R package Hmisc (https://cran.r-project.org/web/packages/Hmisc/) was implemented to evaluate the correlation between genes, gene modules and metabolite clusters with person correlation coefficient. In particular, the accumulation rate of metabolites in fruits was calculated as follows:

*V_t, n_ = (M_t + 1, n_ - M_t, n_) / (T_t + 1_ - T_t_).*

The symbols in the formula above: *n*: metabolite types; *t*: time point of sampling (the values ranging from 1 ~ 3 in this research); *M*: compound content; *T*: the corresponding post-flowering time to each sampling point.

### MYB and GST gene family analysis

The MYB gene and GST genes in *R. tomentosa* and *P. guajava* was identified with hmmsearch based PF00249 from Pfam (https://www.ebi.ac.uk/interpro/) and the functional of eggNOG, respectively. In order to further analyse the phylogenetic relationship and identify the subfamily of the gene in these two families, phylogenetic trees were constructed by iQtree [60]. Based on the functional annotation information, genes from the two families in *A. thaliana* were provided to sequence alignment and further phylogenetic tree construction as the basis for subfamily classification.

### The extraction of flavonoids related compounds

Biological samples were freeze-dried in a vacuum freeze-dryer (Scientz-100F). Freeze-dried samples were crushed with a mixing mill (MM 400, Retsch) and zirconia beads at 30 Hz for 1.5 min. Lyophilized powder, 50 mg, was dissolved with 1.2 mL 70% methanol solution, vortexing for 30 seconds every 30 minutes for a total of six times. After centrifugation at 12000 rpm for 3 min, the extract was filtered (SCAA-104, 0.22 μm pore size, ANPEL, Shanghai, China, http://www.anpel.com.cn/) and then subjected to UPLC-MS analysis.

### Quantification, characterization, and variation analysis of metabolites

The process of quantification and characterization of flavonoids-related metabolites in *R. tomentosa* was based on the protocol in previous research [55]. The k-means algorithm was implemented for the clustering of metabolite content. The cascadeKM function in R packages vegan was utilized to identify the most appropriate cluster number [61].

### Determination of starch and total anthocyanins

Fruit among developmental stages was collected, weighed, and extracted by constant shaking with 1 ml of 100% methanol chilled overnight at 4°C. After centrifugation with 1300 tpm for 10 mins, 200 μL of the supernatant was aspirated and data were recorded at A650 and A666 wavelengths. In addition, 200 μL of supernatant was absorbed for anthocyanin measurement, concentrated hydrochloric acid was added at 1% volume, and then thoroughly mixed. The data were recorded at wavelengths A530 and A657, respectively, and the formula ((A530–0.25*A 657)/fresh weight) was used to calculate the value of anthocyanin [56]. Starch extraction and quantitative detection were performed by Solarbio Life science BC0700 kit (Beijing, China).

## Supplementary Material

Web_Material_uhad005

## Data Availability

The genome assembly as well as the raw genomic data of Illumina sequences, PacBio sequences, ONT sequences and transcriptome data have been deposited in the NCBI Sequence Read Archive under accession number PRJNA893855.
