# Gap-free genome assembly and comparative analysis reveal the evolution and anthocyanin accumulation mechanism of *Rhodomyrtus tomentosa*

**DOI:** 10.1093/hr/uhad057

**Published:** 2023-01-19

This is a correction to: Fangping Li, Shiqiang Xu, Zitong Xiao, Jingming Wang, Yu Mei, Haifei Hu, Jingyu Li, Jieying Liu, Zhuangwei Hou, Junliang Zhao, Shaohai Yang, Jihua Wang, Gap-free genome assembly and comparative analysis reveal the evolution and anthocyanin accumulation mechanism of *Rhodomyrtus tomentosa*, Horticulture Research, Volume 10, Issue 3, March 2023, uhad005, https://doi.org/10.1093/hr/uhad005

In the originally published version of this manuscript, in Figure 4, panels B and C were erroneously transposed.

In addition, in Figure 5B, ‘TJN-4-TJN-gene-131.252’ in the GST-F section was incorrectly rendered in black.

The Publisher apologizes for not correcting these errors at an earlier production stage.

