# Supplementary material for: Gap-free genome assembly and comparative analysis reveal the evolution and anthocyanin accumulation mechanism of Rhodomyrtus tomentosa
Source: Hortic Res. 2023 Jan 19;10(3):uhad005. doi: 10.1093/hr/uhad005 (PMC10022486; doi:10.1093/hr/uhad005)
Supplement: Web_Material_uhad005 [file web_material_uhad005.zip › Revision.Rmspmaterialsub.pdf]

Supplementary material:

Gap-free genome assembly and comparative analysis revealed the differentiation and anthocyanin accumulation mechanism of *Rhodomyrtus tomentosa*

Fangping LI[1, 2+] Shiqiang Xu[1+], Zitong Xiao[1, 2+], Jingming Wang[1, 2], Mei Yu[1], HaiFei Hu[3], Iingyu LI[1], Jieyin Liu[2], Zhuangwei Hou[2], Junliang Zhao[3], Shaohai Yang[1], Jihua Wang[1*]

[1]Guangdong Provincial Key Laboratory of Crops Genetics and Improvement, Crop Research Institute, Guangdong Academy of Agriculture Sciences, Guangzhou 510640, China

[2]Guangdong Provincial Key Laboratory of Plant Molecular Breeding, State Key Laboratory for Conservation and Utilization of Subtropical Agro-Bioresources, South China Agricultural University, Guangzhou 510642, China.

[3]Rice Research Institute & Guangdong Key Laboratory of New Technology in Rice Breeding & Guangdong Rice Engineering Laboratory, Guangdong Academy of Agricultural Sciences, Guangzhou 510640, China.

*Corresponding author: Jihua Wang (wangjihua@gdaas.cn)

Supplementary Table:

Supplementary Table.1 Sample collection and sequencing information

Tiusses	Seq (Llibrary)-types	Data volume
Leafs (Young)	ONT	11019133488
Leafs (Young)	Pacbio-HIFI	33395991644
Leafs (Young)	Hi-C	59451912000
Leafs (Young)	Short fragment (Survey)	66673875600
Friuts (30 day after Flower; F1)-1	RNA-seq/UP-MS	8131029000
Friuts (30 day after Flower; F1)-2	RNA-seq/UP-MS	8564517900
Friuts (30 day after Flower; F1)-3	RNA-seq/UP-MS	7708290300
Friuts (60 day after Flower; F2)-1	RNA-seq/UP-MS	7230245100
Friuts (60 day after Flower; F2)-2	RNA-seq/UP-MS	6937563000
Friuts (60 day after Flower; F2)-3	RNA-seq/UP-MS	7218191700
Friuts (75 day after Flower; F3)-1	RNA-seq/UP-MS	7332127800
Friuts (75 day after Flower; F3)-2	RNA-seq/UP-MS	8852990400
Friuts (75 day after Flower; F3)-3	RNA-seq/UP-MS	8058619500
Friuts (90 day after Flower; F4)-1	RNA-seq/UP-MS	6843585300
Friuts (90 day after Flower; F4)-2	RNA-seq/UP-MS	8061466800
Friuts (90 day after Flower; F4)-3	RNA-seq/UP-MS	9530129700
Root-1	RNA-seq	6560642400
Root-2	RNA-seq	6974283000
Root-3	RNA-seq	6545950500
Flower-1	RNA-seq	6368306100
Flower-2	RNA-seq	6699354600
Flower-3	RNA-seq	6218314200
Stem-1	RNA-seq/UP-MS	8347407900
Stem-2	RNA-seq/UP-MS	7143697500
Stem-3	RNA-seq/UP-MS	6565001400
Old Leafs-1	RNA-seq	7476777300
Old Leafs-2	RNA-seq	6773234100
Old Leafs-3	RNA-seq	8273742900
Old Stem-1	RNA-seq	6706819200
Old Stem-2	RNA-seq	7278009000
Old Stem-3	RNA-seq	6416777700
Leafs (Young)-1	RNA-seq/UP-MS	6913479900
Leafs (Young)-2	RNA-seq/ UP-MS	8872320000
Leafs (Young)-3	RNA-seq /UP-MS	6612349200

Supplementary Table. 2 Telomere positions of *R. tomentosa*

Chromosomes	Telomeres type	Start	end
Chr01	head	1	19031
Chr01	Tail	45748348	45760345
Chr02	head	1	14605
Chr02	Tail	39161264	39171969
Chr03	head	1	9237
Chr03	Tail	43787954	43802537
Chr04	head	1	13929
Chr04	Tail	36177763	36185570
Chr05	head	1	17438
Chr05	Tail	38167592	38177685
Chr06	head	1	18711
Chr06	Tail	50106633	50111023
Chr07	head	1	10390
Chr07	Tail	36351065	36360045
Chr08	head	1	8252
Chr08	Tail	49555476	49565102
Chr09	head	1	8090
Chr09	Tail	41112687	41129775
Chr10	head	1	15573
Chr10	Tail	40112228	41129775
Chr11	head	1	8338
Chr11	Tail	49946678	49958455

Supplementary Table. 3 The distribution of centromere in *R. tomentosa*

Chr	Start	End	CE_start	CE_end	CE_length
Chr1	0	45760552	19269411	19922111	652700
Chr2	0	39171969	15680797	16320794	639997
Chr3	0	43802537	23281852	25569751	2287899
Chr4	0	36185570	19882797	22351168	2468371
Chr5	0	38177685	16305053	18978374	2673321
Chr6	0	50111023	17491730	18317219	825489
Chr7	0	36360045	20178984	20999609	820625
Chr8	0	49565102	25040703	28538774	3498071
Chr9	0	41129775	12638838	15209448	2570610
Chr10	0	40127537	17880905	21271310	3390405
Chr11	0	49958455	22161546	22,514,401	352855

Supplementary Table. 4 Busco of *R. tomentosa* assembly

Statistics	Number (eudicots_odb10)	Number (embryophyta_odb10)
Complete BUSCOs (C)	2271	1598
Complete and single-copy BUSCOs (S)	2230	1578
Complete and duplicated BUSCOs (D)	41	20
Fragmented BUSCOs (F)	13	11
Missing BUSCOs (M)	42	5
Total BUSCO groups searched	2326	1614
Summary	C:97.7%[S:95.9%,D:1.8%], F:0.6%,M:1.7%,n:2326	C:99.0%[S:97.8%,D:1.2%], F:0.7%,M:0.3%,n:1614

Supplementary Table. 5 Types and distribution of TE element

Type	Class	Count	bpMasked	%masked
	Copia	46482	34748751	7.39%
LTR	Gypsy	53608	103546831	22.01%
	unknown	56492	51939143	11.04%
	CACTA	20582	6046185	1.29%
	Mutator	60441	20665458	4.39%
TIR	PIF_Harbinger	11785	3291266	0.70%
	Tc1_Mariner	2842	750050	0.16%
	hAT	14974	5008115	1.06%
nonTIR	helitron	46547	13522456	2.87%
Total		313753	239518255	50.92%

Supplementary Table. 6 Busco of *R. tomentosa* annotation

Statistics	Number
Complete BUSCOs (C)	2168
Complete and single-copy BUSCOs (S)	2088
Complete and duplicated BUSCOs (D)	80
Fragmented BUSCOs (F)	86
Missing BUSCOs (M)	72
Total BUSCO groups searched	2326
Summary	C:93.2%[S:89.8%,D:3.4%],F:3.7%,M:3.1%,n:2326

Supplementary Table.7 Results of the genes in co-expression modules of WGCNA

Supplementary Table.8 The results of compound content clustering and relative abundance.

Supplementary Table.9 The correlation of orthologous genes associated with Anthocyanin synthesis with MYB family in module24 among *R. tomentosa* and *P. guajava*

**Gene in *R. tomentosa***	**Gene in *P. guajava***	***r***	**P**
RmCHI-1	*PgCHI-1*	-0.25237	0.6294822
RmCHI-1	*PgCHI-2*	-0.277897	0.5938857
RmCHI-2	*PgCHI-3*	-0.347461	0.4997829
RmF3H	*PgF3H*	-0.412544	0.4162903
RmDFR	*PgDFR*	-0.518441	0.2920116
RmANS	*PgANS*	-0.509826	0.3015183
TJN_8_processed_gene_9.293	Pgu06979	0.2755447	0.5971433
TJN_7_TJN_gene_159.41 (*RmPAP-1*)	Pgu24212	-0.37864	-0.280597
TJN_7_TJN_gene_159.38 (*RmPAP-2*)	Pgu24788	-0.205914	-0.301088

TJN_1_TJN_gene_136.242	Pgu18946	-0.418678	-0.418678

Supplementary Table.10 The MYB genes with positive selection and subfamily classification

Geneid	MYB Sub-family	Alt	Null	Pvalue
TJN_11_TJN_gene_6_202	MYB65	-4548.30513	-4564.408441	1.38618E-08
TJN_11_processed_gene_25_182	MYB117	-2989.798405	-3038.232554	7.40956E-23
TJN_9_TJN_gene_68_39	MYB86	-3268.165169	-3283.342521	3.59833E-08
TJN_7_TJN_gene_159_38	PAP2/MYB90	-2141.358409	-2180.81265	6.50548E-19
TJN_12_processed_gene_134_36	MYB113	-3601.010041	-3621.604816	1.38159E-10
TJN_7_TJN_gene_123_191	MYB80	-2125.422791	-2136.046978	4.03452E-06
TJN_7_TJN_gene_142_80	MYB80	-2333.496325	-2386.200363	9.94039E-25
TJN_7_TJN_gene_157_71	C8uniq	-3972.098103	-3984.303559	7.78266E-07
TJN_1_TJN_gene_130_23	MYB36	-2102.169767	-2134.840441	6.29858E-16
TJN_3_TJN_gene_30_47	C6uniq	-3236.055744	-3264.477371	4.7197E-14
TJN_10_TJN_gene_131_2	C6uniq	-1892.978145	-1930.22005	6.11376E-18
TJN_4_TJN_gene_131_55	ETC2	-4537.854512	-4557.010291	6.03044E-10
TJN_6_processed_gene_100_163	MYBC1	-3219.713713	-3240.507256	1.12735E-10
TJN_1_TJN_gene_148_217	MYBC1	-2917.379634	-2930.224891	4.00792E-07
TJN_3_TJN_gene_35_156	C6uniq	-2134.848552	-2151.144565	1.1368E-08
TJN_6_TJN_gene_29_140	ETC2	-4451.357965	-4500.100515	5.42631E-23
TJN_6_TJN_gene_115_28	ETC2	-4414.520367	-4505.476903	1.85238E-41
TJN_6_processed_gene_98_76	C7uniq	-1835.617268	-1857.811675	2.69221E-11
TJN_4_processed_gene_25_101	MYBC1	-4207.04195	-4258.26413	4.43655E-24
TJN_10_TJN_gene_107_135	MYBC1	-3465.653519	-3489.101892	7.48268E-12
TJN_10_TJN_gene_123_58	C6uniq	-2970.355217	-3001.355891	3.43222E-15
TJN_1_TJN_gene_144_51	MYBC1	-4590.358497	-4621.578465	2.74696E-15

Supplementary Table.11 The correlation between Myricetin/Dihydromyricetin and its potential synthetic genes.

**Gene**	**Metabolites**	***r***	**P**
RmFLS-1	Myricetin	0.4302945	0.39439352
RmFLS-1	Dihydromyricetin	0.253588	0.627771714
RmFLS-2	Myricetin	0.8889026	0.01782833
RmFLS-2	Dihydromyricetin	0.7591393	0.08003421
RmF3.5.H-1	Myricetin	0.9115781	0.011382
RmF3.5.H-1	Dihydromyricetin	0.9926239	8.14089E-05

Supplementary Figure

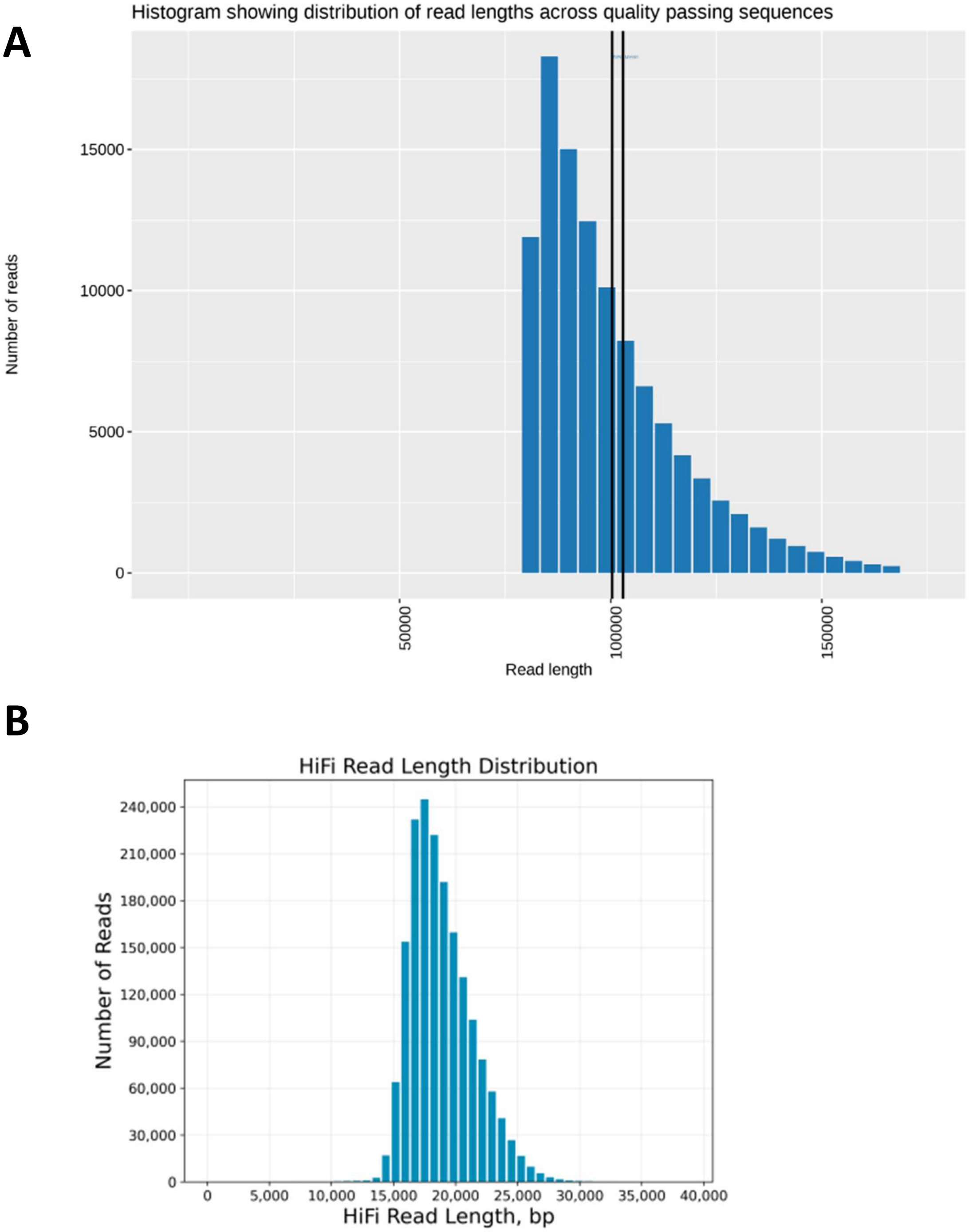

Supplementary Figuer. 1 Distribution of reads length generated by ONT and Pacbio HIFI A: For ONT **B:** For HIFI

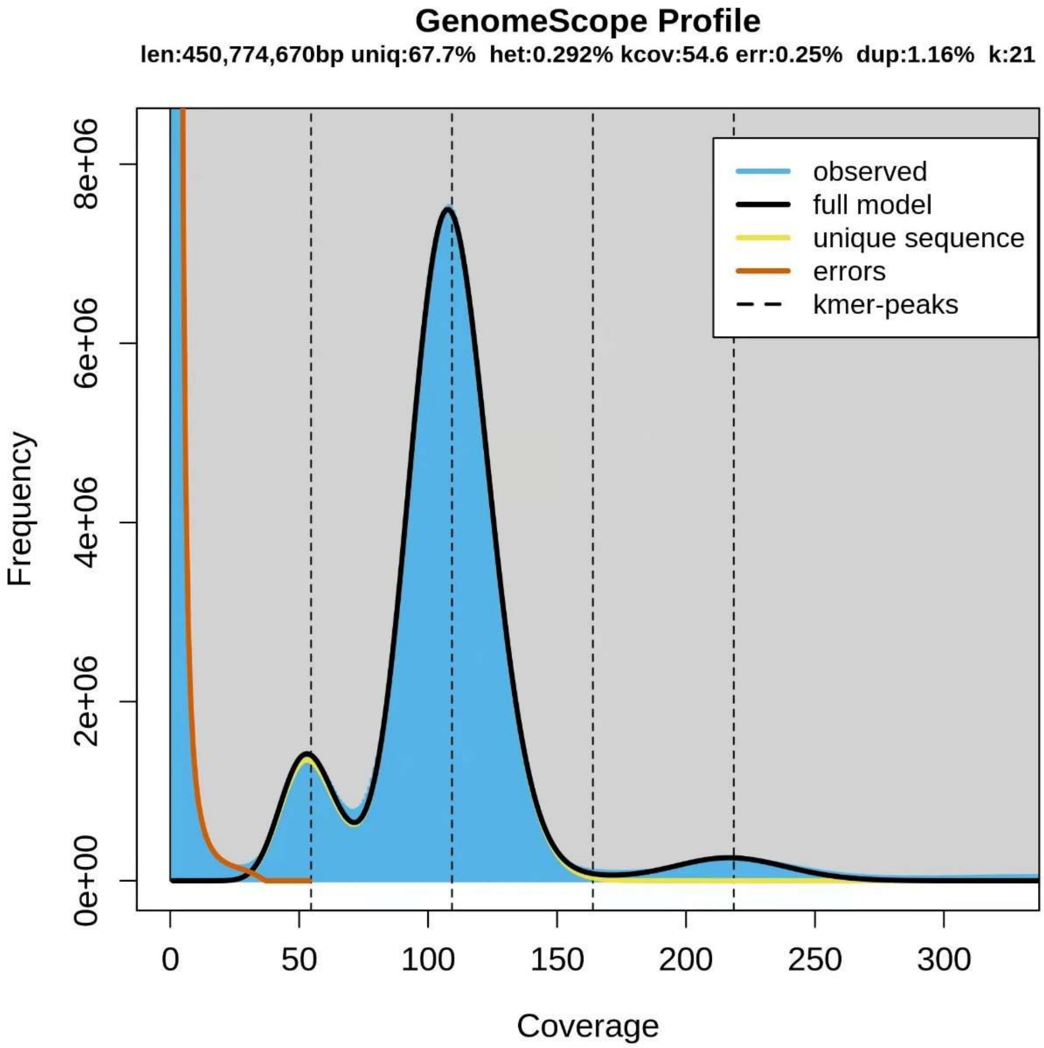

Supplementary Figuer.2 Genome Survey result of *R. tomentosa*

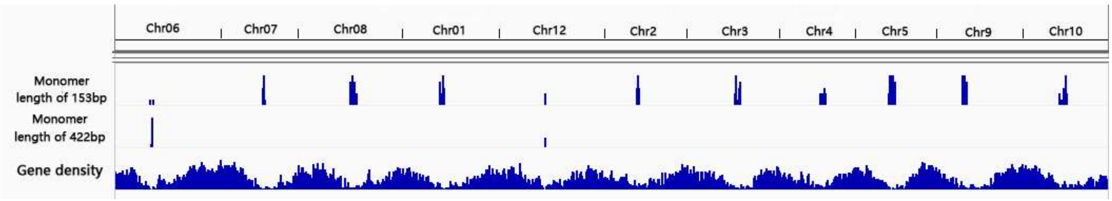

Supplementary Figuer.3 The of distribution of two kinds of candidate centromere tandem repeats and gene density in the ranges of centromere

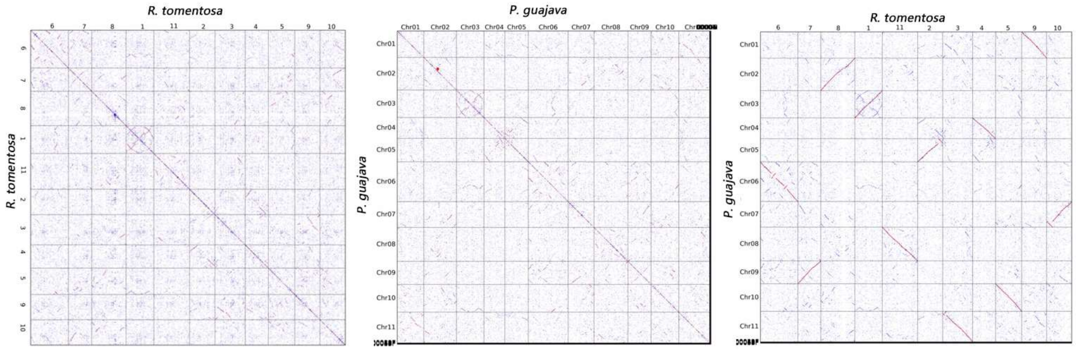

Supplementary Figuer.4 Characteristic of collinearity including *R. tomentosa vs R. tomentosa; P. guajava vs P. guajava; R. tomentosa vs P. guajava;*

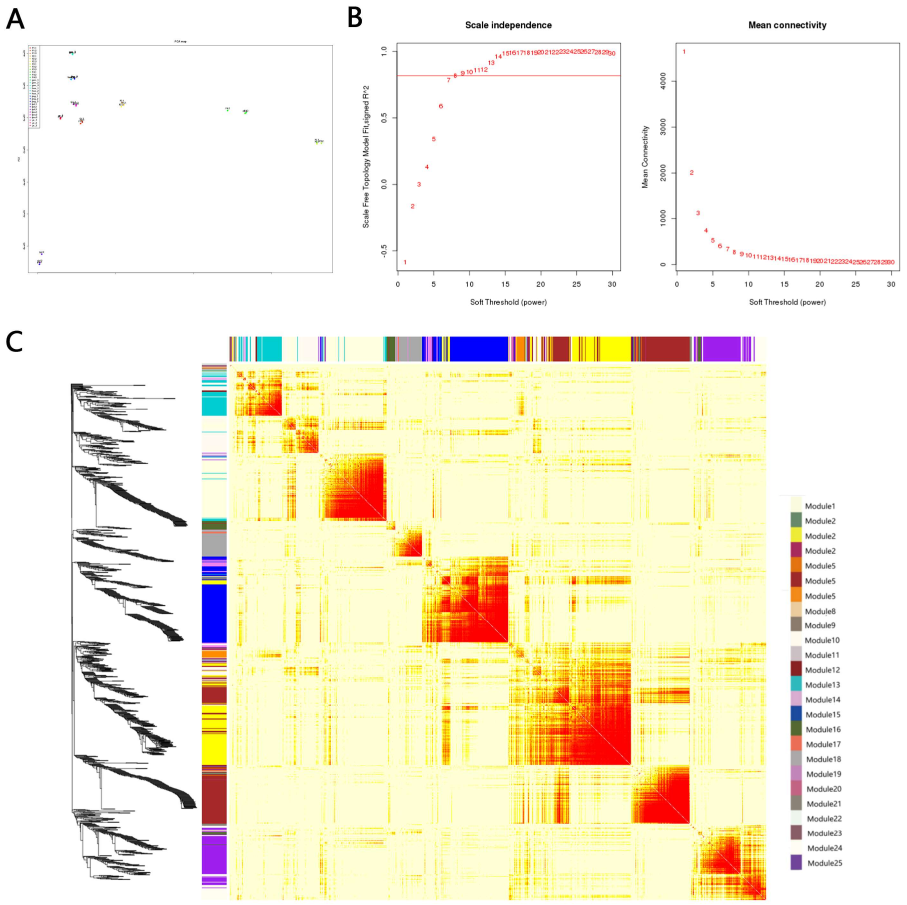

Supplementary Figuer.5 PCA and Weighted-gene co-expression network of transcriptome analysis

A: PCA analysis among the transcriptome samples in this research. **B:** The R^2 distribution among various soft threshold (power) and the mean connectivity, which provided foundation for threshold selection in WGCNA. **C:** Hierarchical cluster dendrogram constructed by WGCNA, on which each leaf represents a gene. 25 merged modules (based on a threshold of 0.20) identified by weighted-gene co-expression network.

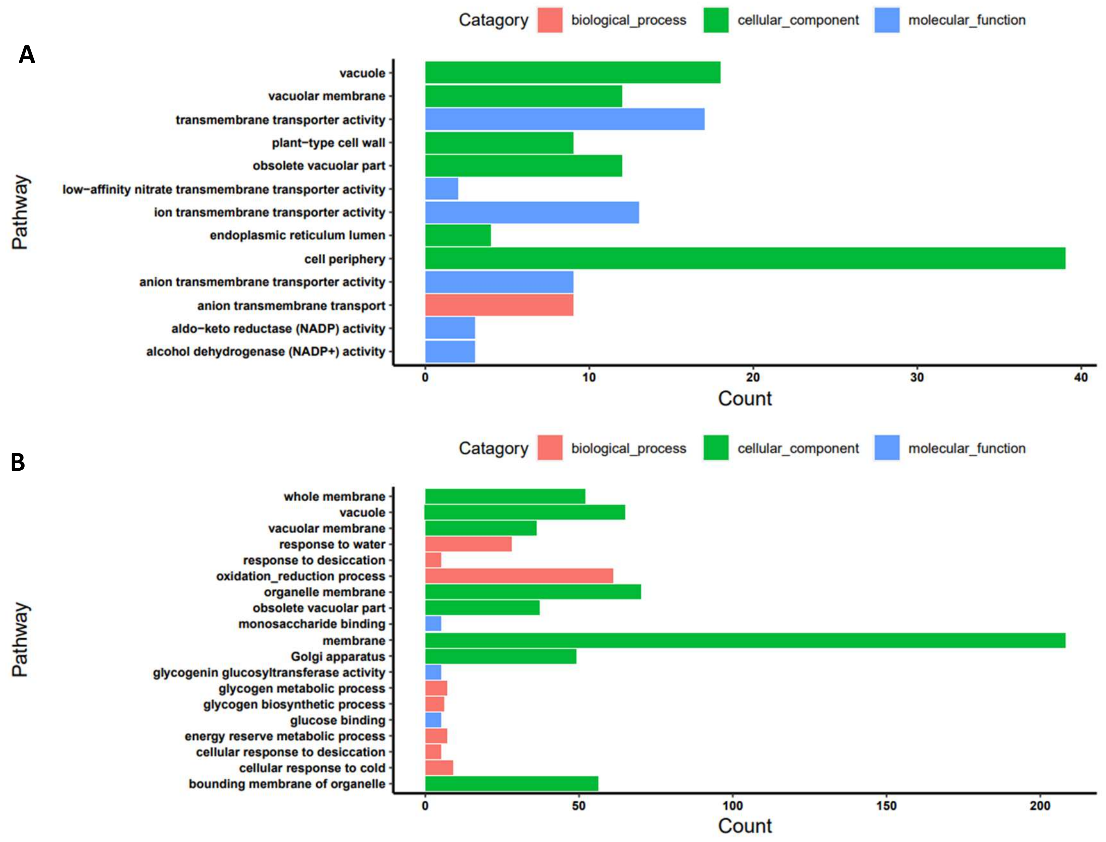

Supplementary Figuer.6 The Go enrichment of the genes in Module16 and Module 25; the ontology with P. adj < 0.05 was displayed;

A: For Module 16 **B:** For Module 25

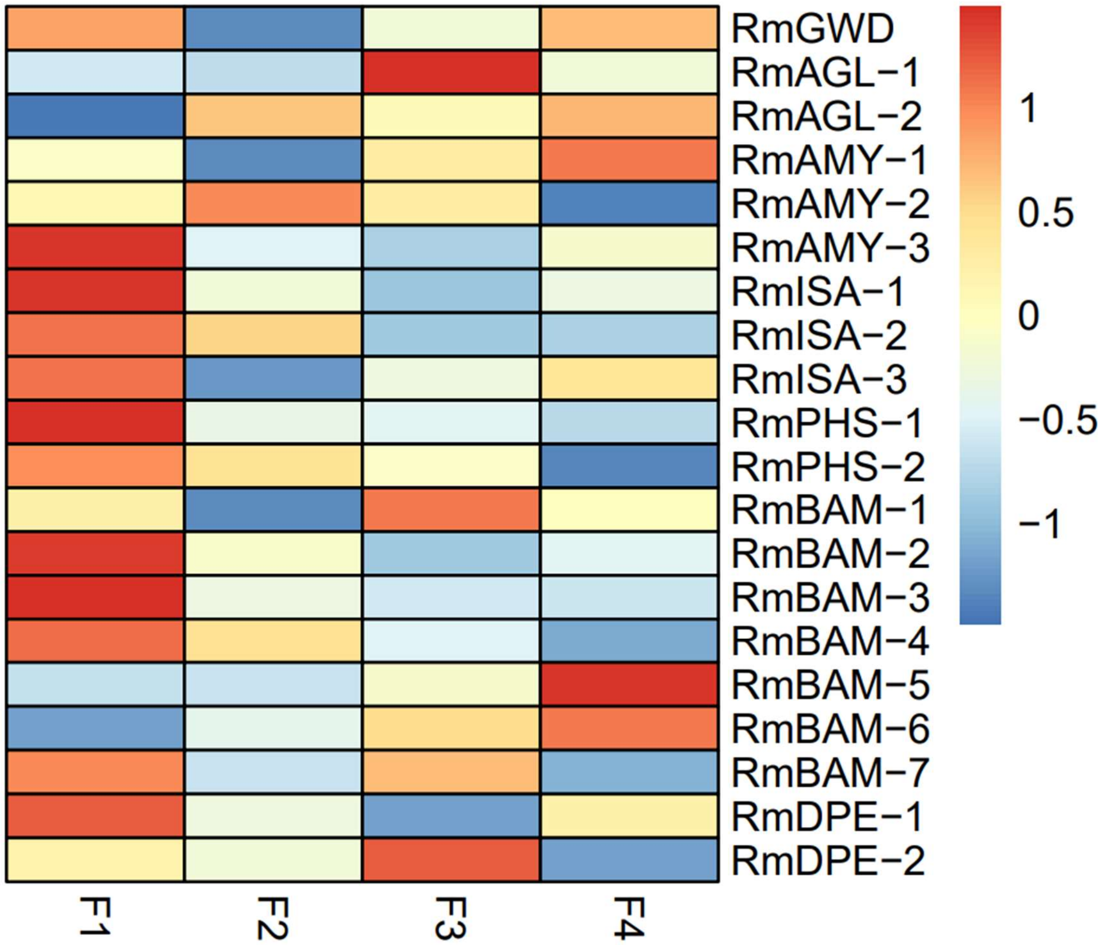

Supplementary Figuer.7 Expression of starch degradation pathway genes

The expression was normalized only for the samples from fruits developmental stages

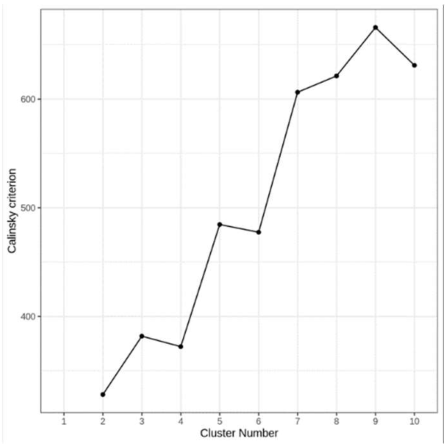

Supplementary Figuer. 8 Calinski Harabaz score distribution

Criteria of Calinski-Harabasz were utilized in the selection of cluster number. The clustering number of Calinski Harabaz score (Calinsky criterion) with the maximum value (K=9; Distribution lines are shown below) for further analysis.

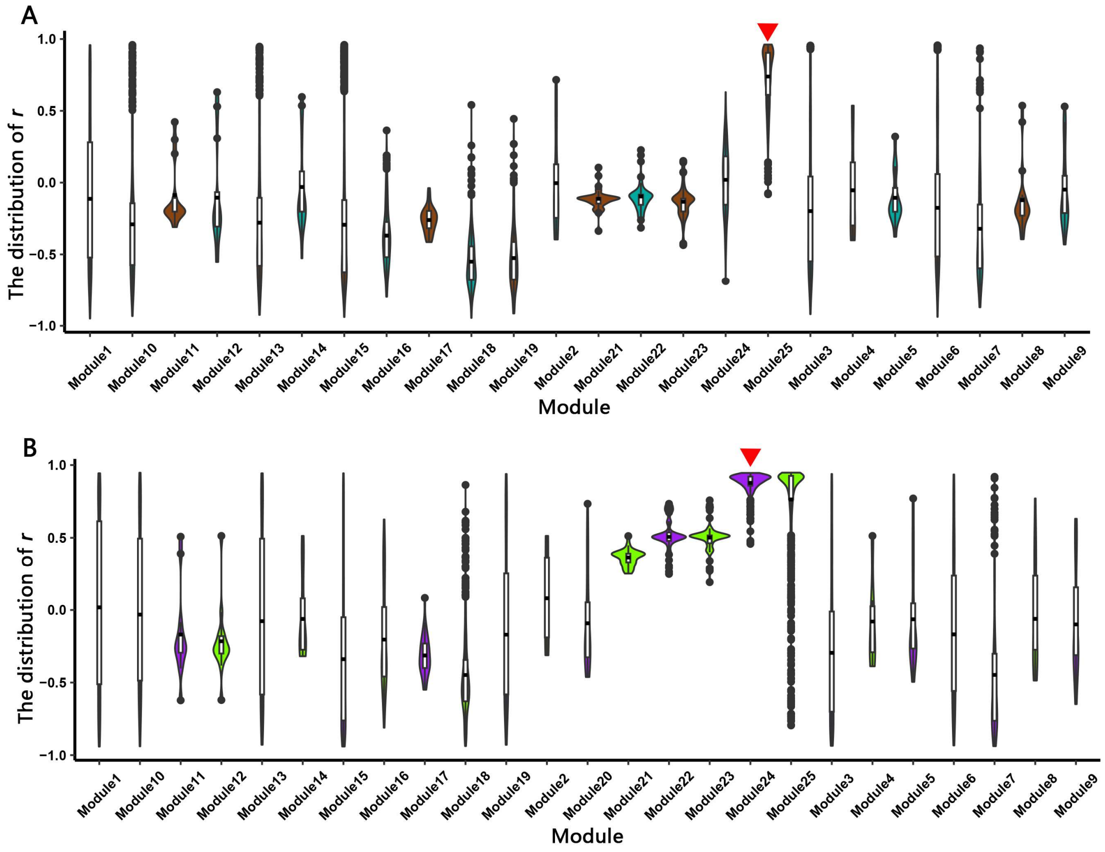

Supplementary Figuer. 9 Correlation distribution between metabolites clustered in cluster8 and modules generated from WGCNA; The modules with the highest average correlation (Pearson correlation coefficient) were marked by red triangles and implemented to further research **A:** For the metabolites content **B:** For the accumulation rate of metabolites

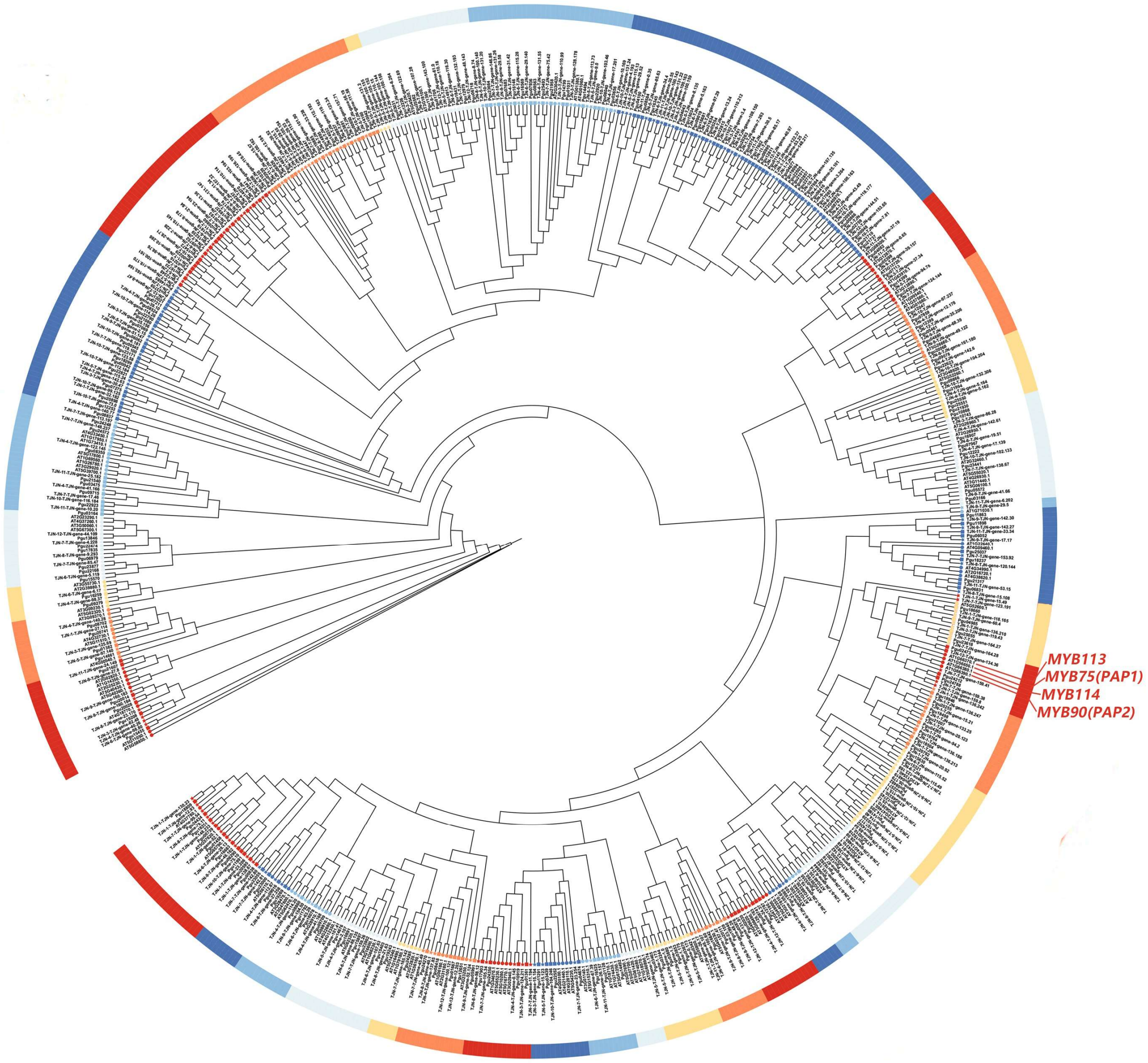

Supplementary Figuer.10 Phylogenetic analysis and gene classification of MYB gene family.

The various colors in outer ring represent the MYB subfamily classification based on *A. thaliana*
